# 3D Bioprinted Spheroidal Droplets for Engineering the Heterocellular Coupling between Cardiomyocytes and Cardiac Fibroblasts

**DOI:** 10.34133/2021/9864212

**Published:** 2021-12-28

**Authors:** Raven El Khoury, Naveen Nagiah, Joel A. Mudloff, Vikram Thakur, Munmun Chattopadhyay, Binata Joddar

**Affiliations:** ^1^Inspired Materials & Stem-Cell Based Tissue Engineering Laboratory (IMSTEL), The University of Texas at El Paso, El Paso, TX 79968, USA; ^2^Department of Metallurgical, Materials, and Biomedical Engineering, M201 Engineering, The University of Texas at El Paso, 500 W. University Avenue, El Paso, TX 79968, USA; ^3^Department of Molecular and Translational Medicine, Center of Emphasis in Diabetes and Metabolism, Texas Tech University Health Sciences Center, 5001 El Paso Drive, El Paso, TX 79905, USA; ^4^Border Biomedical Research Center, The University of Texas at El Paso, 500 W. University Avenue, El Paso, TX 79968, USA

## Abstract

Since conventional human cardiac two-dimensional (2D) cell culture and multilayered three-dimensional (3D) models fail in recapitulating cellular complexity and possess inferior translational capacity, we designed and developed a high-throughput scalable 3D bioprinted cardiac spheroidal droplet-organoid model with cardiomyocytes and cardiac fibroblasts that can be used for drug screening or regenerative engineering applications. This study helped establish the parameters for bioprinting and cross-linking a gelatin-alginate-based bioink into 3D spheroidal droplets. A flattened disk-like structure developed in prior studies from our laboratory was used as a control. The microstructural and mechanical stability of the 3D spheroidal droplets was assessed and was found to be ideal for a cardiac scaffold. Adult human cardiac fibroblasts and AC16 cardiomyocytes were mixed in the bioink and bioprinted. Live-dead assay and flow cytometry analysis revealed robust biocompatibility of the 3D spheroidal droplets that supported the growth and proliferation of the cardiac cells in the long-term cultures. Moreover, the heterocellular gap junctional coupling between the cardiomyocytes and cardiac fibroblasts further validated the 3D cardiac spheroidal droplet model.

## 1. Introduction

Despite the ease of forming and maintaining 2D cultures, some key limitations like failure to mimic the *in vivo* microenvironment and the absence of important cell-cell and cell-matrix interactions, signal transduction, proliferation, and differentiation potential are still a concern [1]. While 2D cultures are most commonly used in *in vitro* research, they are inadequate in fully mimicking the native myocardium and its vascularization, preventing the identification of potential therapeutic targets for cardiovascular diseases [2]. Technical challenges in terms of controlled cell distribution and vascular innervation make *in vivo* transplantation of tissue engineered constructs a rate-limiting step [3]. Three-dimensional (3D) additive manufacturing (AM) technologies offer an exceptional control for design of constructs with complex architectural facets especially in the case of 3D biological tissues.

3D bioprinting is the process of adopting a complex architectural design as found in 3D tissues and translating it into a construct using AM techniques that utilizes biomaterial scaffolds, cells, and growth factors to produce engineered constructs that can serve as physiological replicates of their *in vivo* counterparts [4]. Different combinations of 3D bioprinting techniques and biomaterial scaffolds have been used for tissue engineering overall [5]. 3D *in vitro* multicell systems can not only mimic and represent the *in vivo* environment [5, 6] but can also serve as a promising alternative to animal models to study cardiac developmental biology and recapitulate disease [7]. 3D spheroidal droplets are considered to be the most widely accepted models for 3D *in vitro* culture [8–10]. A scaffold with a spheroidal design is expected to provide an enhanced habitat for tissue formation as it enables sufficient distribution of oxygen, media, growth factors, nutrients, and ions into the scaffold for maintaining cell growth and proliferation [11].

In recent published work, our laboratory has robustly demonstrated the potential of naturally derived hydrogels including gelatin, alginate, and collagen as naturally derived biodegradable materials for bioprinting multilayered systems [12–18]. Sodium alginate, primarily derived from seaweed and exhibiting ionotropic cross-linking mechanism with calcium chloride, sustains bioprinted cell morphology and their functions [11, 19]. The combination of sodium alginate with gelatin improves the ease of printing and its ability for cell anchorage due to the presence of cell adhesion moieties like arginylglycylaspartic acid- (RGD-) containing peptides [20, 21]. Recent studies have also shown the supporting effects of these polymers on sustained release of extracellular matrix (ECM) proteins and growth factors secreted by the cells [22–24].

In this study, we implemented a unique droplet-based extrusion printing technique to 3D bioprint cardiac spheroidal droplets using a CELLINK bioprinter which was scaled up to a higher throughput 96-well array fabrication using the Advanced BioAssemblyBot (BAB), equipped with a six-axis robotic arm. The printing parameters for developing these bioprinted cell 3D spheroid models were optimized, and the printed constructs were physically characterized for their mechanical integrity and structural stability through rheological and swelling studies. Cardiomyocytes and cardiac fibroblasts were premixed in the hydrogels and bioprinted to generate 3D cardiac spheroidal droplets. The resultant cell-based droplets were analyzed for cell viability, and the onset of heterocellular coupling was quantified for up to 21 days *in vitro* [16]. Since the full extent of cardiomyocyte-fibroblast electrical coupling in the heart, the mechanisms of its regulation, and its importance in health and disease have garnered recent substantial interest [25], we propose that these 3D bioprinted cardiac spheroid models can potentially be used in studying and improving the role of heterocellular gap junctional coupling in the transfer of cardiomyogenic signals from the cells within the spheroidal droplets for drug screening or regenerative engineering applications. We further expect the bioprinted 3D spheroidal droplets to be more economical and serve as an enhanced high-throughput platform for functional cardiac assays compared to the flat multilayered systems established earlier in our laboratory [12, 13, 16–18, 22].

## 2. Results

### 2.1. Design, Morphology, and Consistency of 3D Spheroidal Droplets

**Figure 1:**
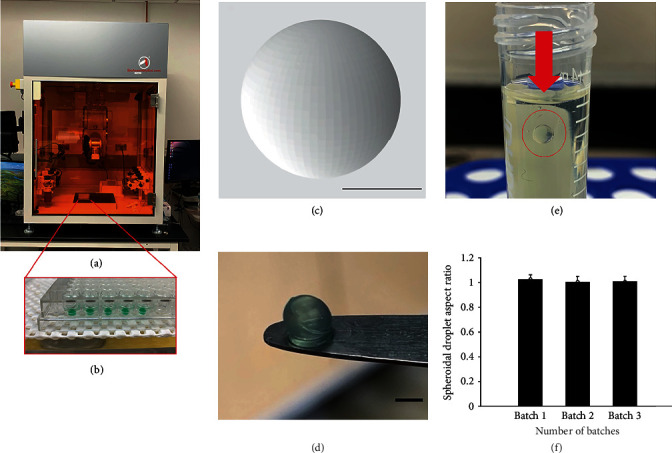
Design, morphology, and consistency of 3D spheroidal droplets. (a) The BioAssemblyBot (BAB) 3D printer used in bioprinting spheroidal hydrogels in a 96-well plate as shown in (b). (c) .stl file image of a spheroidal droplet. (d) Gross morphology of the 3D bioprinted spheroidal droplet. (e) Representative image showing a 3D bioprinted spheroidal droplet freely suspended in oil. (f) Aspect ratio is maintained for three consecutive 3D bioprinted batches. Scale bar was set at 1 mm for (c) and (d).

Figure 1(a) shows the 3D printer used for high-throughput printing of the spheroidal droplets (Figure 1(b)). Figure 1(c) depicts the structural design adopted for printing the scaffolds. Figure 1(d) represents the 3D printed spheroidal droplets obtained from a gelatin- and sodium alginate-based bioink. The optimized printing parameters for obtaining stable scaffolds using the CELLINK and the BAB platforms are summarized in Table 1. The objective of the parameter optimization was to reduce the shear stress acting on the bioink to obtain maximum geometric accuracy [13, 15]. The various parameters for 3D printing were modulated to achieve geometric accuracy by maintaining the extrusion pressure constant due to its inverse effect on cell viability. This optimization process for printing of 3D spheroids resulted in four distinct generations (Gen I-III) prior to yielding the desired 3D cardiac spheroids (Gen IV). Corresponding results for each generation are in Supplementary Table S1.

The partial ionic cross-linking of the sodium alginate in the hydrogel mixture with different concentrations of calcium chloride solution was found to be beneficial in obtaining structurally stable 3D bioprinted hydrogels. Gelatin was not cross-linked before printing; however, it is expected that the bioink mixture containing gelatin and alginate would lead to physical entanglements between the two polymeric chains resulting in electrostatic interactions leading up to partial ionic cross-linking between these two subunits (Supplementary Table S1 and Figure S1). Suspension of the 3D bioprinted spheroidal droplets in castor oil confirmed its morphological and structural stability as depicted in Figure 1(e). The aspect ratio of the 3D bioprinted hydrogel spheroidal droplets was maintained between varying batches. Figure 1(f) represents the aspect ratio of the 3D bioprinted spheroidal droplets (Gen IV) over three batches from a total of 108 samples suggesting its consistency and reproducibility of the spheroidal prints using the in-house developed printing technique.

### 2.2. Microstructural Analysis

**Figure 2:**
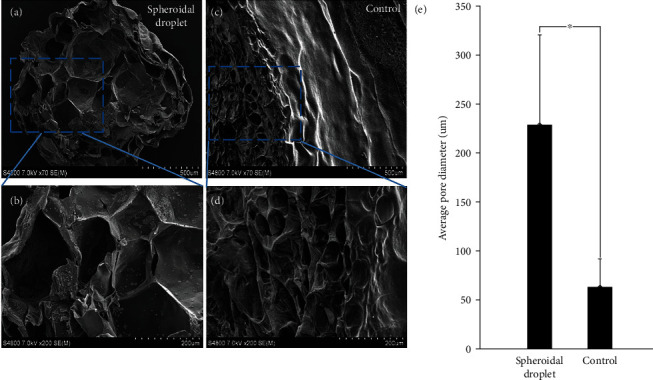
Microstructural analysis. (a, b) Representative SEM images for the cross section of the spheroidal droplets. (c, d) Representative SEM images for the cross-sectional structures of the flattened disk structure (control). (e) Graph depicting average pore diameter of the spheroidal droplet in comparison to the control structure. At least 5 representative images were acquired for analysis to generate the data in (e). Average pore diameter was found to be statistically different (^∗^*p* < 0.05) between the spheroidal droplet and control samples.

Figures 2(a)–2(d) represent the microstructural cross section of the 3D spheroidal droplets (a, b) and flattened disks (c, d) performed using scanning electron microscopy (SEM). Both spheroidal droplets and flat disks exhibited a porous and interconnected network allowing their use as 3D cell culture models [26]. However, a significant difference in average pore diameters of the 3D bioprinted spheroidal droplets and flattened disks (controls) (*p* < 0.001) was observed and depicted in Figure 2(e). Porosity in a hydrogel scaffold plays an important role in cell survival, proliferation, and migration while pore interconnectivity influences cell ingrowth, vascularization, and nutrient diffusion for cell survival [27]. Engineered tissue requires pore sizes greater than 200 *μ*m for rapid vascularization and survival [28]. A significantly higher average pore diameter was observed for 3D bioprinted hydrogel spheroidal droplets with respect to the control samples (*p* < 0.001). The average pore diameter of the 3D spheroidal droplet was 228.67 ± 92.07 *μ*m while the controls exhibited an average pore diameter of 63.02 ± 29.3 *μ*m (*p* < 0.001). A higher pore diameter contributes to a significantly higher porosity thereby increasing the interconnectivity between the pores which may enable and facilitate higher cell migration and diffusion of nutrients, supplements, and oxygen more efficiently in the 3D spheroidal droplets compared to the control structures [12, 29]. Interestingly, this also sheds light on the possible mechanisms of the droplet printing in comparison with flattened disks. Since the 3D spheroidal droplets are extruded as a single droplet deposited within one well and cross-linked instantaneously, this leads to scaffolds with high porosity that are associated with higher mass transport of oxygen and nutrients, facilitating tissue regeneration [30]. On the other hand, the flattened disks have decreased average pore size and are associated with an increase in the surface area and suffer from decreased mass transport of oxygen and nutrients as well.

### 2.3. Swelling and Mechanical and Structural Properties of Bioprinted Constructs

**Figure 3:**
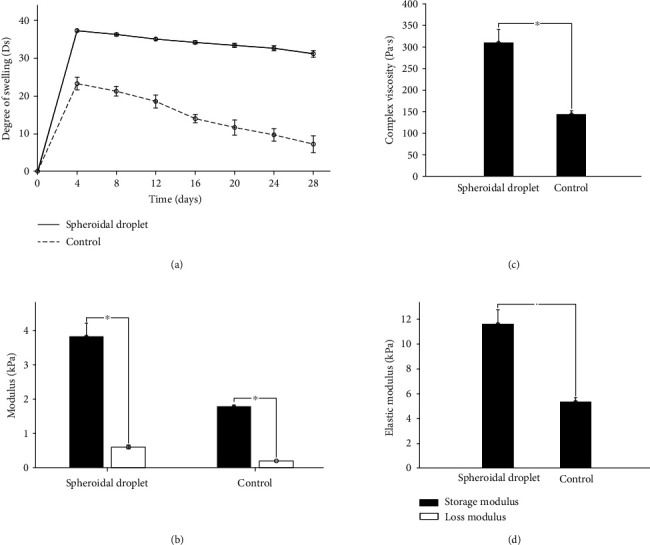
Mechanical characterization. (a) Graph depicting average degree of swelling (mean ± SD) for spheroidal droplets (black) and controls (blue) for a period of 28 days. (b) Graph showing the storage modulus and loss modulus of the spheroidal droplet in comparison to control. (c, d) Graph showing the complex viscosity and the elastic modulus of the spheroidal droplet in comparison to control. At least three repeats (*n* = 3; 3 × 24 spheroidal droplets and 3 × 1 for flattened disk) were used for calculation. ^∗^*p* values were found to be all statistically different.

Figure 3(a) shows the degree of swelling (*D*_s_) of the 3D spheroidal droplets in comparison to the flat disk control samples. 3D bioprinted hydrogel spheroidal droplets had a significantly higher degree of swelling that was nearly twice compared to controls (*p* < 0.01). For both samples, the maximum swelling ratio was observed after 24 hours from when samples remained stable throughout the duration of the experiment up to 28 days. The higher surface to volume ratio combined with the higher average pore diameter and open-closed pore ratio in 3D spheroidal droplets contributed to increased swelling compared to controls [31]. It is to be noted that the spheroidal droplets were stable till 28 days in 1x PBS and did not physically disintegrate while the control samples started physically disintegrating after 14 days. This confirms that the 3D spheroidal droplets can be utilized for long-term *in vitro* cell culture studies.

Figures 3(b)–3(d) represent the viscoelastic properties of the 3D spheroidal droplets and the control samples. While both sets of samples exhibited mainly elastic properties, the storage and loss modulus of the 3D spheroidal droplets and the control samples varied significantly as shown in Figure 3(b) (*p* < 0.001 between the spheroidal droplets and controls). The average storage/loss modulus for the 3D spheroidal droplets was 3.84 ± 0.38/0.597 ± 0.054 kPa while control structures exhibited 1.78 ± 0.05/0.188 ± 0.002 kPa for storage and loss moduli, respectively. Similar to the storage and loss modulus, the elastic modulus of 3D bioprinted hydrogel spheroidal droplets was significantly higher than the control samples (*p* < 0.001). The storage modulus represents the energy stored in the elastic structure of the sample whereas the loss modulus represents the viscous part or the amount of energy dissipated in the sample [32, 33]. If storage modulus is higher than the loss modulus, the material can be regarded as mainly elastic and vice versa. The elastic modulus, which is a measure of the scaffold's resistance to being deformed elastically, was also studied. The higher pore diameter and interconnectivity of the 3D spheroidal droplets in comparison with the controls may have manifested in higher sets of values for storage/loss and elastic moduli [25]. Thus, the degree of elasticity was greater in the 3D spheroidal droplets in comparison with the controls. The elastic modulus of the spheroidal scaffolds is within the range of the elastic modulus of native heart tissue (>10-15 kPa) making it an attractive platform to be used as 3D cardiac organoids for *in vitro* studies. Although this data does not recapitulate values with cell content, it is estimated that inclusion of cells would yield higher values of elastic moduli which would pose as excellent choices for cardiac scaffolds used in tissue engineering.

Complex viscosity, which is a measure of the total resistance to flow as a function of angular frequency [34], was measured in both 3D spheroidal droplets and controls (Figure 3(c)). The 3D spheroidal droplets had a significantly higher complex viscosity (Figure 3(c)) (309.78 ± 30.86 Pa · s) than controls (143.11 ± 8.58 Pa · s).

### 2.4. Cell Viability within the Spheroidal Droplets

**Figure 4:**
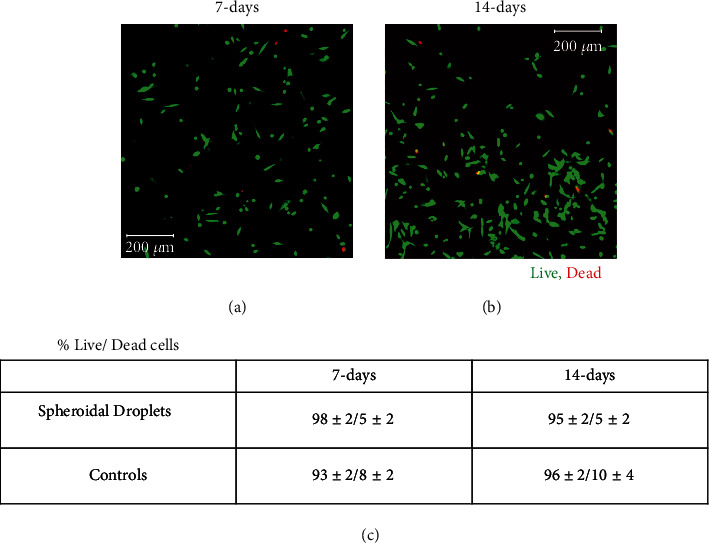
Cell viability. (a, b) The live stained cells in green by calcein AM and dead cells stained in red by ethidium homodimer after 7 days and 14 days in culture (scale bar 200 *μ*m). (c) % live/dead cells in both 3D spheroidal droplets and controls at 7 and 14 days, respectively. *p* values were found not to be statistically significant when data was compared between different time points.

Figures 4(a) and 4(b) represent the live-dead confocal microscopy images of cells in the 3D spheroidal droplets after 7 and 14 days, respectively. More than 90% of cell viability was detected through the live-dead assay performed in the cross-sectional core of the scaffold.  Figure 4(c) shows no significant difference in the % of live cell viability between the two time points representing the steady turnover of the growing number of the cells in both scaffolds, spheroidal droplets and flat disks. Since no significant change in cell viability was noted at increasing time points, the mechanical stability of the scaffolds was robust enabling the viability of the cells. Live-dead images from other controls are depicted in Supplementary Figure 2.

In order to confirm if a necrotic core was formed in the center of the 3D spheroidal droplets containing cells, SEM and TUNEL assay was performed en face on the cross section of samples cut through the center at day 7 to corroborate the viability data obtained from imaging (as in Figure 4). Supplementary Figures 3A and 3B represent the SEM images of the 3D cell spheroidal droplets, and Supplementary Figure 3C represents the elemental analysis of the scaffolds with and without cells. The SEM images (S3A-B) revealed the encapsulation and distribution of cells within the spheroidal droplets. Moreover, we identified and located multiple possibly viable cells within the spheroidal structure, as seen from their adherence to the scaffold (yellow arrows in S3A). The elemental analysis obtained through energy-dispersive spectra (EDS) of the cell laden and acellular scaffolds shows a significant difference in C : N : O ratio between acellular and cell-laden 3D bioprinted hydrogel scaffolds signifying the presence of cells in the spheroidal droplets.

**Figure 5:**
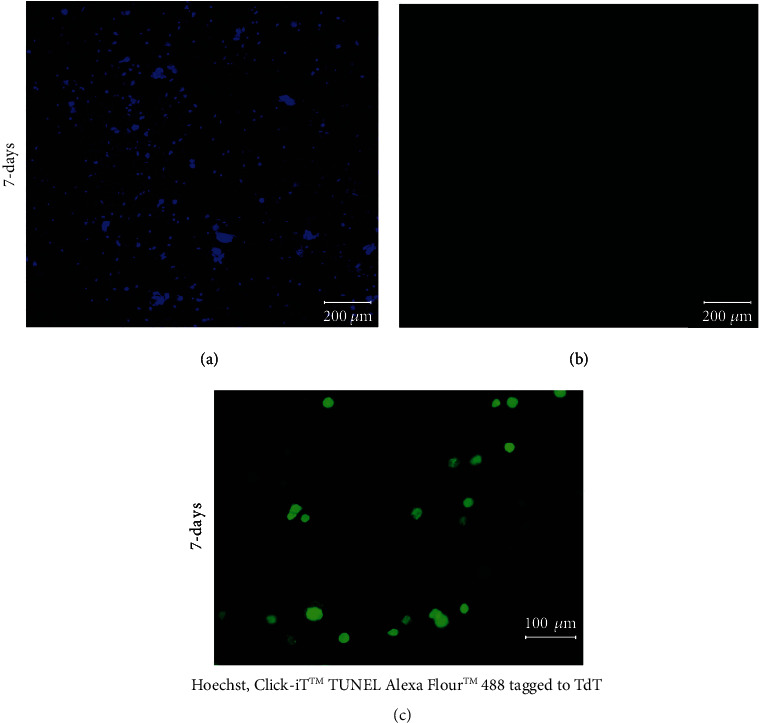
TUNEL staining of 3D spheroidal droplets. (a) All the cells counterstained using Hoechst and (b) the absence of dead cells after 7 days in culture (scale bar 200 *μ*m) imaged from the same sample section. (c) The presence of dead cells in the 3D spheroidal droplets.

Furthermore, TUNEL assay results depicted in Figures 5(a)–5(c) confirm the absence of a necrotic core in the cardiac spheroidal droplets. When 3D cardiac spheroidal droplets cultured in vitro for 7 days were processed using the Click-iT™ TUNEL Alexa Fluor™ 488 tagged to the enzyme terminal deoxynucleotidyl transferase (TdT), no evidence of apoptotic DNA fragments could be detected (Figure 3(b)) when all cells were counterstained using Hoechst (Figure 3(a)). However, a parallel set of samples treated with ethanol for 5 min and then processed using the TUNEL assay revealed positive staining for the Alexa Fluor™ 488 tagged to TdT (Figure 3(c)). This observation was corroborated also by the live/dead assay results in cells printed in a 3D spheroid stained in red by ethidium homodimer after intended apoptosis with ethanol treatment (Supplementary Figure S2C).

### 2.5. Heterocellular Coupling of CFs with CMs and Phenotypic Characterization

**Figure 6:**
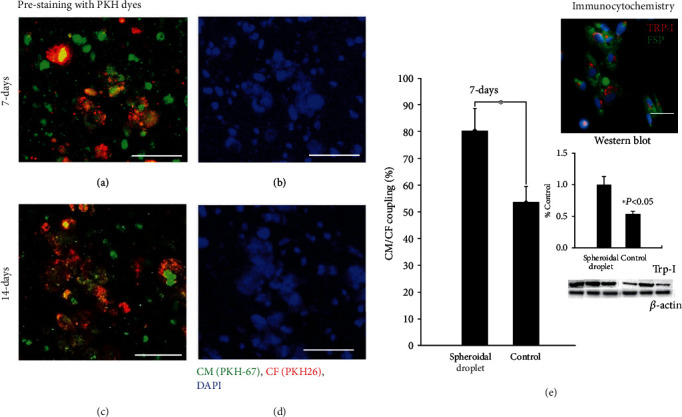
Confirmation of heterocellular coupling for cardiomyocyte (CM) and cardiac fibroblasts (CF) in the spheroidal droplets. Heterocellular coupling between CM (green) and CF (red) is shown after 7 and 14 days of culture (a–d). % of heterocellular coupling is depicted using a bar graph in (e). The extent of coupling was found to be significantly greater in the 3D spheroidal droplets (*p* < 0.03) compared to the controls after 7 days of culture. Immunocytochemical data for CM-CF coculture at 7 days demonstrated the presence of CM and CF as analyzed by cell marker troponin I for CM and FSP for CF and imaged as shown in (e). Scale bar in all images is 100 *μ*m. Furthermore, Western blot analysis demonstrated an increase in Trp-I expression in the spheroidal droplets compared to the controls, *p* < 0.05. Trp-I bands for protein expression are depicted along with a row of *β*-actin that was used as a housekeeping control protein. Bands were obtained by running 25 *μ*g of protein, and membrane was developed using 1 min exposure.

In an attempt to mimic *in vivo* cardiac tissue, the 3D spheroidal droplets must promote heterocellular coupling (HC) between cardiomyocytes and fibroblasts [22]. Representative high-magnification images of the PKH-prestained CMs and CF in 3D spheroidal droplets after 14 days are depicted in Figures 6(a)–6(d). The cells confirmed their respective identities as CM and CF with heterocellular and cell junction coupling between the cells as shown in Figure 6(e) (top right corner). Troponin I (Trp-I: red) was used as a biomarker for staining the CM while fibroblast-specific protein I (FSP-I: green) was used as a biomarker for the CF. DAPI (blue) was used as an overall nuclear stain for all cells depicted in Figure 6. The enmeshing of both cell types, CM and CF (Figures 6(a)–6(d)), clearly confirmed their HC within the 3D spheroidal droplets at early and later time points in his study. The heterocellular coupling is consistent and maintained even after 21 days of culture as shown in Supplementary Figure S4. Figure 6(e) (bar graph) depicts the quantitative analysis of percentage HC between the 3D spheroidal droplets and controls (flattened disk images not included). The percentage of HC was 80.33 ± 8.38% (*p* < 0.05) for the 3D spheroidal droplets and 53.63 ± 5.9% for controls further emphasizing the potential of using these 3D bioprinted spheroidal droplets as cardiac organoids. The extent of HC (%) was confirmed to be within the range of studies published previously by our group [16].

Furthermore, Western blot analysis (as shown in Figure 6(e) bottom right corner) demonstrated a decrease in Trp-I (24 kDa) expression in the controls in comparison to the spheroidal droplets (*p* < 0.05). This confirms the increased cell coupling and the presence of an increased density of cells in the spheroids which possibly leads to the enhanced expression of Trp-1 in these samples.

### 2.6. Cell Proliferation

**Figure 7:**
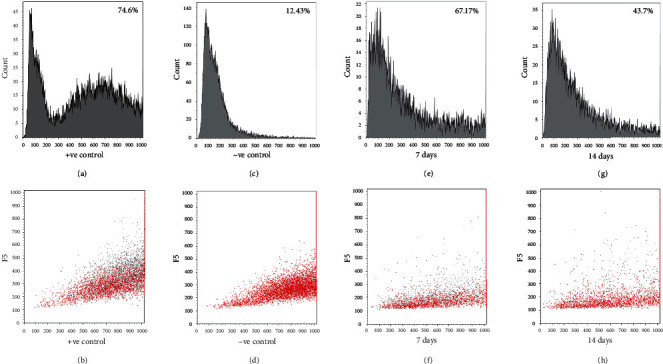
Cell proliferation analysis. CM cells were prestained with CellTrace Violet (CTV) and mixed with CF (1 : 1) for bioprinting within spheroidal droplets and cultured for long term *in vitro*. At 7 days and 14 days of culture, all cells were extracted from the spheroidal droplets and assessed using a FACS analyzer. Shown in (a, c, e, g) are representative graphs depicting % of CTV dye intensity (relates to the overall quantity of cells stained using CTV) for each time point and each condition. Positive control indicates CM cells prestained with CTV and mixed with CF (1 : 1) for bioprinting within spheroidal droplets and cultured for only 24 hours after which they were extracted. Negative control indicates the exact conditions for positive control without the addition of the CTV dye. Shown in (b, d, f, h) are the representative scatter plots showing the red dots (unstained CF) and grey dots (CTV-stained CM in all cases except −ve control) for each of the conditions depicted. In (a, c, e, g), the *y*-axis depicts “counts,” and in (b, d, f, h), the *y*-axis depicts “FS” or forward scatter plots.

We were specifically interested to study the proliferation trends for CM when they are coupled with the CF. Figures 7(a)–7(h) represent the flow cytometry analysis of cells encapsulated in the 3D spheroidal droplets. The CMs were stained using CellTrace™ Violet (CTV) dye, and the CFs were unstained. The CTV dye was used to track cell growth and proliferation using the concept of dye dilution and was used to investigate cell proliferation of CMs when coupled with CF in a 1 : 1 ratio in the 3D spheroidal droplets after 7 and 14 days of culture. This concept permits several generations of proliferating cells to be examined. Thus, increasing cell proliferation causes a reduction in intensity of the dye over successive generations produced [35, 36]. In this experiment, positive controls included CMs prestained with CTV and mixed with CF (1 : 1) for bioprinting within 3D spheroidal droplets and cultured for only 24 hours after which they were extracted. Negative controls included samples cultured using exact conditions as positive controls, without the addition of the CTV dye.

Figure 7(a) represents characteristic peaks from positive controls wherein peaks for unstained CF are observed in the low-intensity region in the left (<300) and peaks for stained CM with high intensity is observed in the right (>300). The corresponding scatter plot for Figure 7(a) is depicted in Figure 7(b) where grey dots depict the CM and red dots depict the CF. Figure 7(c) represents characteristic peaks from negative controls where no cells were stained and shows peaks only in low-intensity regions in the left (<300). The corresponding scatter plot for Figure 7(c) is depicted in Figure 7(d). These characteristic peaks displayed by both sets of controls can be further explained by the higher intensity in the positive control (74.6%) when compared to negative control (12.43%) at day 1. Figures 7(e)–7(h) represent the percentage (%) of the dye intensity collected from samples extracted after 7 and 14 days of culture, respectively. The % intensity of the CTV dye decreased from 67.17% at day 7 to 43.7% at day 14. This reduction in intensity of the dye from the range > 300 clearly signified the dilution of dye with time indicating proliferation of CM in the 3D spheroidal droplets. In addition, it is noteworthy to mention that the reason that the CM peaks were seen to be flattening out after 14 days of culture is because they were being outnumbered by CFs which grow more robustly and at a faster rate [37].

Moreover, the presence of multiple peaks (Figures 7(e) and 7(g)) indicates the creation of new generations of cells during their incubation period. The corresponding scatter plots for Figures 7(e) and 7(g) are depicted in Figures 7(f) and 7(h). All trends were statistically significant (*p* < 0.001).

Confocal microscopy images for CTV dye-stained cell samples are presented in the supplementary section (Figure S5).

## 3. Discussion

In recent years, 3D structures composed of multiple cells such as spheroidal droplets and organoids have been utilized extensively in *in vitro* research. Spheroidal droplets are 3D simple cell aggregates of broad-ranging cells, such as from tumor tissue [38], embryoid bodies [39], hepatocytes [40], nervous tissue [41], or mammary glands [42]. Cell-based clusters or cell spheroidal droplets are coaxed to form 3D cultures without a scaffold [38–42]. Thus, they cannot self-assemble or regenerate and thus are not as advanced as organoids. On the contrary, organoids are complex clusters of organ-specific cells and self-assemble when given a specific scaffolding extracellular environment, such as Matrigel [43] or collagen [44]. This leads to the development of microscopic versions of parent organs feasible for use as 3D study tissue models.

In this study, we developed a cardiac organoid model using spheroidal droplets for facilitating the heterocellular coupling between cardiac myocytes and cardiac fibroblasts. In the cardiac wall *in vivo*, cardiac myocytes and fibroblasts form extensive networks in the heart, with numerous structural contacts between these two cell types and other supporting cells. The cardiac fibroblasts secrete the majority of the extracellular cardiac tissue matrix, and their number increases with aging and during disease [45]. The cardiac myocytes which are coupled by connexin-43 (Cx43) gap junctions are known to be distinctly separated from fibroblasts *in vivo*. However, *in vitro*, these two heterogeneous cell types form functional gap junctions, which serve as a substrate for electrical coupling of cardiac myocytes [46]. Whether similar behavior occurs *in vivo* has been the subject of considerable discussion. Prior published studies from others including electrophysiological, immunohistochemical, and dye-coupling data confirmed the presence of direct electrical coupling between the two cell types in normal cardiac tissue in the sinoatrial node *in vivo* [47]. In our prior published studies, we have demonstrated proof of *in vitro* heterocellular coupling between cardiac myocytes and cardiac fibroblasts, facilitated by 3D bioprinting *in vitro* [16]. Thus, there is a strong correlation based on such heterogeneous cell coupling behavior, the cardiac electrical impulse conduction, and the transport of small molecules or ions in both the normal and pathological myocardium both *in vitro* and *in vivo*. Interestingly, cardiac fibroblasts have been shown to increase Cx43 expression in experimental models of myocardial infarction. Therefore, in the setting of cardiac disease, enhanced cardiac myocyte and fibroblast heterocellular coupling may influence the electrical activity of the myocytes and contribute to arrhythmias. This heterocellular coupling phenomenon is key to understanding the potential active contribution of nonmyocytes to cardiac electrophysiology and their relevance towards cardiac structure and function.

The key objective in this study was to create a 3D cardiac tissue model that may facilitate the study of biomarkers for targeting therapeutic strategies to treat cardiac disease and thereby allow for a better understanding of cardiac biology. We hypothesized that 3D cardiac cell spheroidal droplets will serve as a platform for enabling the heterocellular coupling between cardiac myocytes and fibroblasts for studying drug interactions in the future. After standardization of the 3D bioprinting parameters, this study resulted in high-throughput production of 3D spheroidal droplets that exhibited interconnected porosity that promoted cell viability and function.

Each 3D spheroidal droplet has a volume of 4.18 mm^3^ and contains 20,000 ± 1000 cells calculated from an initial cell density of 1 million cells per mL (corresponding to 4785 cells/mm^3^). On the contrary, each control structure had a volume of 21.2 mm^3^ and contains 100,000 ± 4000 cells while maintaining cell density constant (corresponding to 4717 cells/mm^3^). Furthermore, from 1 mL of bioink mixed with cells, approximately 45 ± 3 3D spheroidal droplets versus 9 ± 1 flat structures can be printed. The possibility for further improvement of an already well-defined 3D biofabricated cardiac tissue system method was therefore established by showing that an advanced scaffold design may provide cellular diversity and culture efficiency [16]. Thus, the 3D cell spheroidal droplets represented a more efficient and economic model for studying cardiac cellular interactions [48–50]. This observation should remain consistent with any other 3D block structures if printed with cells for comparison with the spheroidal droplets. Furthermore, the spheroidal structures demonstrated a greater percentage of open versus closed pores in comparison to the flat disks. Interconnected networks of open pores are essential for cell nutrition, proliferation, and migration for new tissue formation [27]. Scaffolds with high resultant porosity (e.g., spheroids) enable effective transport of substrates for nutrient exchange. However, the mechanical fidelity of the spheroidal scaffolds was also maintained by a moderate presence of closed pores which lead to balance between the mechanical and mass transport function for an optimal scaffold system.

Like fetal cardiomyocytes, tissue engineered cardiac models also should express an upregulation and expression of cardiac troponin I (Trp-I) and tropomyosin as they transform into in mature cardiomyocytes [51]. Based on this hypothesis, we studied the expression levels of Trp-I in both 3D cell spheroidal droplets and 2D flattened disks. Our hypothesis was confirmed by the upregulation of Trp-I in the 3D cell spheroidal droplets in comparison to the 2D flattened disks.

The model geometry is significantly altered between the two structures, with the spheroidal droplets posing essentially as a 3D unit whereas the flattened disks posed as 2-layered structures. Keeping gel volume constant for both structures, adoption of the two designs used in this study led to differences in degrees of curvature among both scaffolds. This led to alteration in cell behavior as well as also differences in mechanical behavior although both scaffolds were made using the same bioink [17].

Since homogenous delivery of oxygen to 3D static cell cultures remains an unsolved challenge, it can be expected that irregular oxygen supply may impede uniform cellular growth leading to decrease in overall cell viability within the spheroidal droplets over sustained periods, e.g., one month. In future studies, we will adopt dynamic culture conditions to sustain prolonged cell viability in long-term spheroidal cultures.

Our model can be used in high-throughput drug testing and screening processes allowing the automated testing of extensive and broad range of factors and biological compounds on a particular target or biomarker [52]. Nevertheless, the bioprinting protocol parameters and dimensions of the spheroid can be further adjusted to reflect a wider range of *in vivo* tissues and for usage in other applications such as tumor models [53, 54]. On the other hand, drawbacks of such a model arise primarily from not resembling the striated structure of the native heart tissue [55]. Since the dimensions of the spheroid is such that it is made to fit inside a 96 well and since the maximum number of cells that it can hold is around 4 × 10^6^/sample at confluence, effective balancing of initial cell seeding density and scaffold volume must be taken into consideration prior to bioprinting.

In the future, we will adopt this 3D spheroidal model for studying the contractile behavior of cell types such as HL-1 and even present a third cardiac cell type such as endothelial cells that will serve as a complete cardiac cell model applicable towards tissue engineering applications. The 3D spheroidal model is versatile and can be adopted for analysis of cell behavior and function to study the effect of various drugs that are known to impose cardiotoxicity [56, 57]. This model is flexible enough to be adopted for other types of tissue engineering involving other cell types and can be used for microfluidic studies as well.

## 4. Materials and Methods

### 4.1. Chemicals and Cell Culture Reagents

Alginic acid (sodium salt; medium viscosity (MVG), MP Biomedicals, LLC, Illkirch, France) and gelatin type A (MP Biomedicals, LLC, OH, USA) were mixed together to form the hydrogel mixture. Sodium citrate and ethylenediaminetetraacetic acid (EDTA) were procured from Fisher Scientific (USA). Calcium chloride solution was created from calcium chloride dihydrate (Fisher Chemical, Germany) mixed with phosphate-buffered saline 10x solution (Fisher Bioreagents, USA) as an ionic cross-linker for the hydrogel scaffolds. Castor oil USP (Walgreen CO, Illinois, USA) was used to suspend the printed spheroidal droplets for structural analysis.

Cardiac fibroblast complete growth medium (Cell Applications, CA, USA) was used as such to culture CFs, and the CMs were cultured and expanded in Dulbecco's modified Eagle medium (DMEM/F12; Sigma Cat. No. D6434) containing 2 mM L-glutamine (EMD Millipore Cat. No. TMS-002-C), 12.5% FBS (EMD Millipore Cat. No. ES-009-B), and 1x penicillin-streptomycin solution (EMD Millipore Cat. No. TMS-AB2-C). CellTrace Violet proliferation kit (Invitrogen, Carlsbad, CA) and DAPI (Thermo Fisher Scientific, USA) were used for cell labeling. In addition, 96 round-bottom well plates and 24 and 6 flat-bottom well plates (Thermo Fisher Scientific, USA) were used for bioprinting and cell culture, respectively, and Trypsin-EDTA (0.25%, phenol red, Thermo Fisher) for cell detachment; 3-(4,5-dimethylthiazol-2-yl)-5-(3-carboxymethoxyphenyl)-2-(4-sulfophenyl)-2H-tetrazolium (MTS; G3580, Cell Titer 96 Aqueous One Solution Cell Proliferation Assay, Promega, USA) was used for cell viability assays. The LIVE/DEAD® Viability/Cytotoxicity Kit was acquired from Thermo Fisher to image the extent of viability of cells. Multitissue dissociation kit 1 (Miltenyi Biotec GmbH, Bergisch Gladbach, Germany) was used to enzymatically digest the hydrogels for cell extraction and PFA (paraformaldehyde solution, 4% in PBS, Janssen Pharmaceuticals, Belgium) applied for cell fixation. Click-iT™ Plus TUNEL Assay for In Situ Apoptosis Detection was acquired from Invitrogen™, Thermo Fisher Scientific, USA. Hoechst 33342 was used for counterstaining of cell nuclei (Sigma-Aldrich, St. Louis, MO, USA). Reagents for Western blotting are included in Section 4.10.

PKH26 Red and PKH67 Green, Fluorescent Cell Linker Kit for General Cell Membrane Labeling were acquired from Sigma-Aldrich.

### 4.2. Preparation of Alg/Gel Hydrogels

The bioink solution of 2% *w*/*v* gelatin was dissolved in Milli-Q water (37°C) under constant stirring to which 3% *w*/*v* alginate was added under aseptic conditions [58]. The ink solution was next vortexed for 2 min to dissolve the alginate-gelatin mixture and centrifuged at 1200 rpm for 3 min to remove the remaining air bubbles. Before cell printing, gels were UV sterilized for 15 min after which they were loaded into a 3 mL syringe (CELLINK, Blacksburg, VA, USA).

### 4.3. Fabrication of Bioprinted Constructs

A 3D spheroid of dimensions 2 mm × 2 mm was designed using SolidWorks® software. The scaffold design was then converted to a binary .stl file using Meshmixer®, a prototype designer tool. Using CELLINK BIO X (Blacksburg, VA, USA), the temperature-controlled printer head was used to deposit droplets inside a 96 round-bottom well plate. 5 *μ*L of 80 mM CaCl_2_ sterile solution was preadded to the bottoms of each of the wells. As shown in Table 1, air pressure was set between 13 and 15 kPa, speed selected to be 0.7 mms^−1^, and a nozzle size of 16G (ID = 1.2 mm/OD = 1.6). After printing, alginate-gelatin composite scaffolds were cross-linked with an additional 75 *μ*L of 80 mM CaCl_2_ solution for 45 min under dynamic conditions while being placed on a Belly Dancer Shaker (IBI SCIENTIFIC, Iowa, USA). For high-throughput droplet printing of the hydrogel, BioAssemblyBot was used. An array of spheroidal droplets with identical dimensions was designed using TSIM software, and design file was transferred into the HMI software (Human Machine Interface) connected to the BAB. High-throughput 3D bioprinting was achieved using parameters as mentioned before. A flattened disk was 3D printed using the same parameters [12], served as a control structure in this study. Scaffolds were cross-linked and washed under dynamic conditions with 1x PBS (pH 7.4) for 20 minutes to remove excess calcium chloride. To test the uniformity and the conservation of the overall structure of the 3D spheroidal droplets, three distinct batches (*n* = 36/batch) were printed. Random samples picked from each of these three batches were then submerged in castor oil, and the aspect ratio of each spheroid sample was calculated using ImageJ by finding the relationship between the height and width of each corresponding spheroid generation [59] and depicted in

(1) $$\begin{array}{c} \text{Aspect ratio}=\frac{\text{Height}}{\text{Width}}. \end{array}$$

### 4.4. Scanning Electron Microscopy

En face and cross-sectional images of the previously lyophilized hydrogels were analyzed using a SEM (Hitachi, S-4800, Japan). Samples for imaging were sputter coated with gold for 45 seconds using Gatan Model 682 precision etching coating system, Pleasanton, CA, and imaged between 5 and 10 kV [12, 14]. The elemental analysis of the cross section of 3D bioprinted hydrogel spheroidal droplets with/without cardiac cells was carried out in situ using the energy-dispersive X-ray spectroscopy (EDS) instrument in conjunction with the SEM. The characterization of elemental composition revealed the presence of C, O, N, and Na. The electron beam was focused on cells adhered to scaffold in the core and on the scaffolds alone in case of acellular scaffolds to show the difference in elemental composition as described by other published works [60]. An in-depth microstructural analysis was performed using ImageJ, and average (AVG) pore diameter was calculated utilizing

(2) $$\begin{array}{c} \text{AVG Pore Diameter}=\frac{\sum \text{P}\text{ore Diameters}}{\text{Total No}.\text{of Pores}}. \end{array}$$

Porous scaffolds have a mesh-like structure with open surface pores which are often interconnected in the bulk [61]. On the other hand, closed pores appear as cavities in the scaffolds but are not interconnected with the rest of the scaffold structure [62]. Scaffold porosity is determined by accounting for closed and open pores of varying size, shape, spatial distribution, and mutual interconnection. The greater extent of open porosity has a considerable influence on the physiochemical properties of the scaffold as well as biocompatibility and tissue regeneration.

### 4.5. Swelling and Degradation

The 3D spheroidal droplets and controls were maintained at -80°C for 24 hours and lyophilized using BenchTop Pro with Omnitronics, SP Scientific, PA, USA (-86°C, 205 mT) for another 24 hours [12]. After freeze-drying, samples were weighed (*W*_0_) and immersed in 1x PBS. At various time points, the samples were weighed at room temperature [12, 15]. Swelling ratio was calculated using the formula below for samples equilibrated in PBS for up to at least 28 days.

(3) $$\begin{array}{c} D_{s}=\frac{W_{i}-W_{0}}{W_{0}}. \end{array}$$

### 4.6. Rheological Analysis

To examine the rheological properties of the experimental and control constructs, hydrogels were preswollen in 1x PBS for 24 h before testing. Oscillatory shear stress rheometric study was performed using an Anton-Paar MCR 92 rheometer (Anton-Paar, Austria) with a PP25/S measuring system at 1% strain with a frequency range between 0.5 and 50 Hz [63]. Analysis for frequency and strain was conducted within the viscoelastic range of the gels. Storage/loss moduli, complex viscosity, and elastic modulus were measured at 1.99 Hz as previously done and reported [12–14, 63].

### 4.7. Biocompatibility

Human cardiac fibroblasts (CF, adult; Cell Applications, San Diego, CA) and AC16 human cardiomyocyte or cardiac myocyte cell line (CM, ATCC, Manassas, VA) were mixed in a ratio of 1 : 1 to constitute a final cell seeding density of 1 × 10^6^ cells added to 1 mL of the alginate-gelatin bioink solution and 3D bioprinted to obtain cell laden 3D bioprinted spheroidal droplets and flattened disks as controls. The ratio of 1 : 1 for mixing the cells was adopted from prior published studies wherein the ratio of these cells in the native cardiac tissue was revisited [64]. Cultures were incubated with a complete growth medium made from CF and CM growth media combined together in a ratio of 1 : 1 and maintained at 37°C and at 5% CO_2_. To assess the biocompatibility of the 3D spheroidal droplets, Live/Dead Assay Kit (Thermo Fisher Scientific, USA) was used according to the protocol provided by the manufacturer. Calcein AM (green) represented live cells while ethidium homodimer (red) represented dead cells; viability of cells was quantified using

(4) $$\begin{array}{c} \text{No}.\text{of live}/\text{dead cells }\left(\%\right)=\frac{\#\text{of green or red cells}}{\text{Total no}.\text{of green and red cells }}\times 100. \end{array}$$

To confirm their HC, biomarkers (see Section 4.9) were used in addition to prestaining the cells with PKH dyes, according to the protocol provided by the manufacturer. Briefly, the CMs were stained using PKH67 (green) and the CFs were stained using the PKH23 (red) as previously reported by our group [12–14].

The samples for HC analysis were fixed with PFA for 15 min at room temperature (25-28°C) prior to imaging. The samples were then washed thrice with 1x PBS, mounted using Fluor mount-G with DAPI (Thermo Fisher Scientific). Using an LSM 700 confocal microscope system (Zeiss, Germany), Z-stack projection high-magnification images were acquired for each representative sample in each group and the experiment was repeated at least thrice. The average coupling percentage was obtained using

(5) $$\begin{array}{c} \%\text{Coupling}=\frac{2\times \left(\#\right.\text{ of coupled CMs }\left(\text{green}\right)\&\text{CFs }\left(\text{red}\right)}{\text{Total no}.\text{of CFs}+\text{CMs }}\times 100. \end{array}$$

### 4.8. TUNEL Assay and Imaging

DNA fragmentation in the cells within the 3D bioprinted cardiac spheroidal droplets was determined using Click-iT™ Plus TUNEL Assay for In Situ Apoptosis Detection (Invitrogen™, Thermo Fisher Scientific, USA). After 7 days of culture, 3D spheroidal droplets were fixed in 4% paraformaldehyde for 15 min and then cut in half exposing the core of the scaffold followed by a staining procedure with picolyl azide Alexa Fluor™488 visualizing the DNA degradation according to the manufacturer's protocol [65]. The nuclei were counterstained with Hoechst 33342 (Sigma-Aldrich, St. Louis, MO, USA). The stained sample sections were visualized using an LSM 700 confocal microscope system (Zeiss, Germany) using the appropriate filters. Z-stack projection high-magnification images were acquired for each representative sample in each group, and the experiment was done in triplicates.

### 4.9. Immunohistochemistry

The 3D bioprinted spheroid hydrogel constructs in media were harvested and fixed in 1 : 1 acetone/methanol fixative for 20 minutes at -20°C. This was followed by immunostaining using fibroblast surface protein (FSP-1 for CF, 1 : 400, Sigma), troponin I (for CM, 1 : 400, Thermo Fisher), and DAPI as mentioned previously to identify individual cell types [22, 49]. Goat anti-rabbit IgG secondary antibody-Alexa Fluor 594 or goat anti-mouse IgG secondary antibody-Alexa Fluor 488 conjugate applied at a dilution of 1 : 1000 was used as secondary antibodies. The samples were then washed thrice with 1x PBS, mounted using Fluor mount-G with DAPI (Thermo Fisher Scientific). Using a Nikon Eclipse NiE microscope system (Nikon Instruments Inc., Melville, NY), single-plane high-magnification images were acquired for each representative sample in each group and the experiment was repeated at least thrice [16]. For the % coupling analysis, Z-stack images (3-5 images/sample) were used for the data calculation.

### 4.10. Western Blot

Samples isolated from culture were washed thrice with sterile 1x PBS until they were clear from culture media. Next, the samples were suspended in a solution of sodium citrate and EDTA (1 : 1) at final concentration of 0.05 M each for 15 min at RT. After this step, the gels were cut apart using a cell scraper into smaller fragments and the suspension was homogenized with pipetting. Once the suspension was completely homogenized, it was centrifuged at 400 × *g* for 10 min to collect the cells in a pellet form [66].

To the isolated cell pellet, 2x Laemmli buffer (Bio-Rad Laboratories, USA) containing protease inhibitor cocktail (5x) and dithiothreitol (DTT:100 mM) was added to resuspend the pellet and vortex it; following which the samples were heated at 100°C for 5 min to extract the cellular proteins. Next, the extracted proteins were estimated using a NanoDrop A280 (Thermo Fisher Scientific) that measures absorbance of mainly tryptophan and phenylalanine and provided an overall estimation of the amounts of amino acids in the protein mixture present in our extracts. A total of 25 *μ*g of protein was run in gels (Bolt 4-12% BT Plus, Life Technologies) at 100 V for 2 h. For the transfer, PVDF membranes of 40 *μ*m size were used to capture the proteins in a Mini Blot Module, Life Technologies) at 20 V for 60 min.

After successful transfer of proteins, the membrane was blocked with 5% nonfat milk in 1x TBS-T, rinsed thrice with 1x TBS-T for 5 min each followed by antibody treatment. A primary antibody treatment using Trp-I (Life Technologies, 16A11) was done in a 1 : 500 dilution in 1x TBS-T for 1 h at RT, followed by three washes with TBS-T for 5 min each. Next, the membrane was probed with a secondary antibody solution of goat anti-mouse IgG (H+L) Superclonal™, HRP conjugate based on suppliers' protocols (Life Technologies, A28177) diluted 1 : 4000 in 1x TBS-T by 1 h at RT, washed thrice with TBS-T for 5 min each, and immediately imaged with Pierce™ ECL Western Blotting Substrate (Thermo fisher, PI32209). *β*-Actin (1 : 2000; MilliporeSigma, St. Louis, MO, USA) was used as a loading control, and the data were normalized with the respective level of *β*-actin using an image analysis software (ChemiDoc XRS System, Bio-Rad Laboratories, Hercules, CA, USA) to determine the intensity of each band (*n* = 3 sample per group) after which the data were further analyzed to assess the percent of control.

### 4.11. Flow Cytometry Analysis (FACS)

Cardiomyocytes were prestained using CellTrace™ Violet (CTV) proliferation kit (Invitrogen, Carlsbad, CA) according to the manufacturer's protocol prior to 3D bioprinting with the cardiac fibroblasts in alginate-gelatin hydrogel. To further confirm the uptake and staining of the CTV dye in cells prior to FACS analysis, cells were imaged using an EVOS bright field microscope (Figure S4). The 3D spheroidal droplets with cells were cut using a blade, and cells were extracted using Miltenyi gentleMACS Dissociator (Miltenyi Biotec, Cambridge, MA) using a Multitissue Dissociation Kit-1 by running the Multi_B program according to the manufacturer's protocol. After 7 and 14 days, cells were fixed with 4% PFA for 15 min at room temperature and added to their designated FACS analysis falcon tubes and analyzed using Beckman Coulter Gallios Flow Cytometer (Brea, CA, USA) using excitation and emission wavelengths of 405 and 450 nm, respectively.

### 4.12. Statistical Analysis

All experiments were performed in triplicate with varying passage of cells for both cardiomyocytes and cardiac fibroblasts (passages 8-12) and scaffolds (from at least 3 different batches) for this study, and numerical data are reported as mean ± standard deviation. Each experiment included at least 3 technical replicates and 3 biological replicates. All data were compared using Student's *t*-test with *p* < 0.05 considered to be statistically significant.

## Figures and Tables

**Table 1:** Conditions used for 3D bioprinting of spheroidal droplets.

Generation	Hydrogel composition	Printer head temperature (°C)	Bed temperature (°C)	Printer speed (mm/s)	Extrusion pressure (kPa)	Nozzle size/inner diameter (mm)	Preaddition of CaCl_2_ (*μ*L)	Postaddition of CaCl_2_ (*μ*L)	Average aspect ratio
IV	2% gelatin 3% MVG alginate	Room temperature (25-28°C)	Room temperature (25-28°C)	0.7 mm/s	13-15 kPa	16 G (~1.19 mm)	5 *μ*L	75 *μ*L	1.0124 ± 0.0437 mm

## Supplementary Materials

Supplementary Materials Table S1: the evolutionary process in engineering a 3D bioprinted spheroid. Figure S1: average aspect ratio (mean ± SD) calculations for the other corresponding generations as enlisted in Table S1. Figure S2: cell viability was assessed by performing the live/dead assay on 2D structures after 4 days of culture. Figure S3: scanning electron microscopy and EDS for cellular encapsulation. Figure S4: shown is an image depicting heterocellular coupling between CM (green) and CF (red) after 21 days of culture (left). All cells (CM+CF) in the presented image are depicted with DAPI (blue) staining (right). Figure S5: shown is a bright field image of CTV dye-stained CM cells.
