# Supplementary material for: 3D Bioprinted Spheroidal Droplets for Engineering the Heterocellular Coupling between Cardiomyocytes and Cardiac Fibroblasts
Source: Cyborg Bionic Syst. 2021 Dec 28;2021:9864212. doi: 10.34133/2021/9864212 (PMC9254634; doi:10.34133/2021/9864212)

Graphical Abstract

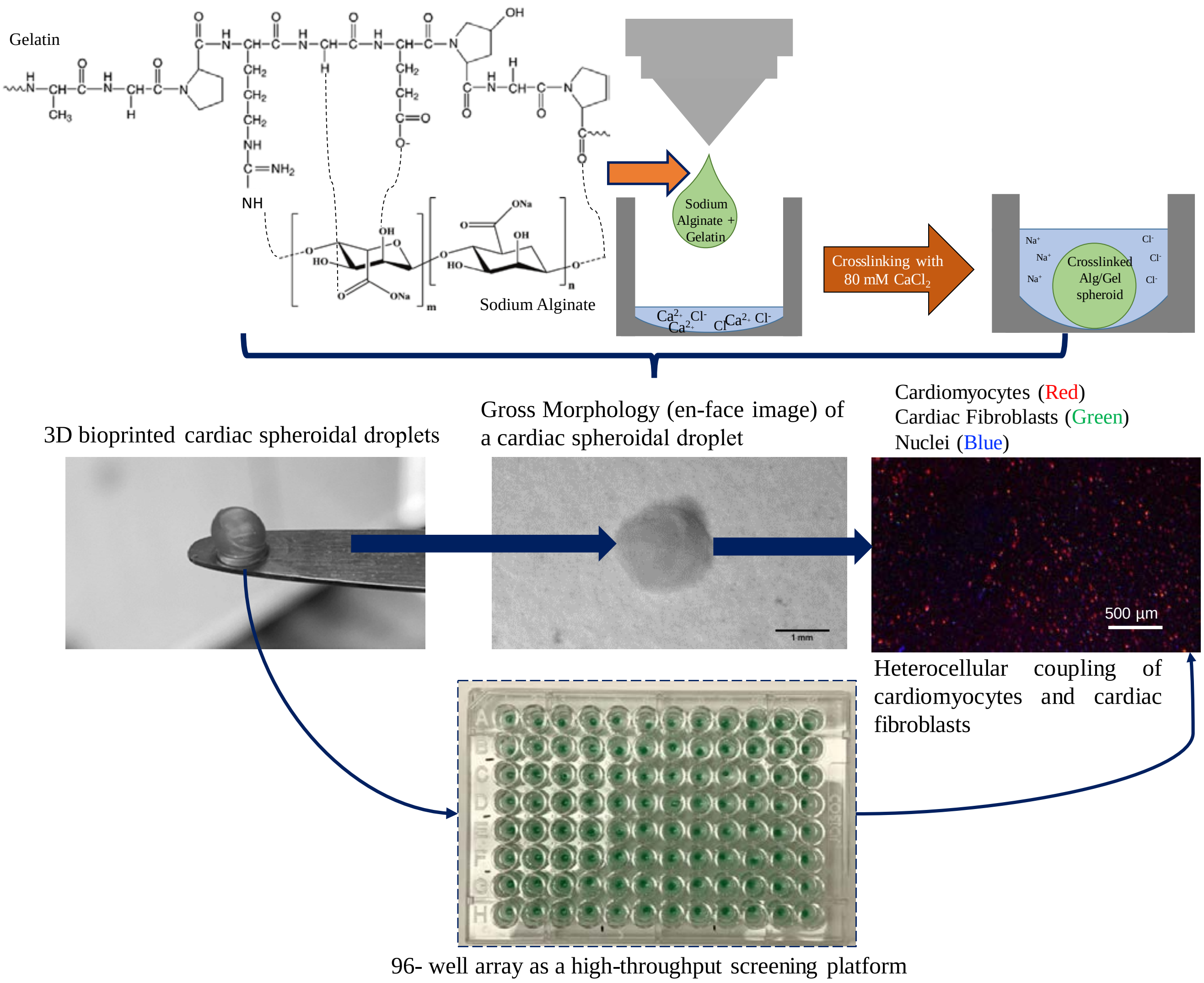

Supplement: Supplementary Materials — Table S1: the evolutionary process in engineering a 3D bioprinted spheroid. Figure S1: average aspect ratio (mean ± SD) calculations for the other corresponding generations as enlisted in Table S1. Figure S2: cell viability was assessed by performing the live/dead assay on 2D structures after 4 days of culture. Figure S3: scanning electron microscopy and EDS for cellular encapsulation. Figure S4: shown is an image depicting heterocellular coupling between CM (green) and CF (red) after 21 days of culture (left). All cells (CM+CF) in the presented image are depicted with DAPI (blue) staining (right). Figure S5: shown is a bright field image of CTV dye-stained CM cells. [file 9864212.f1.zip › GA.pdf]
