# Supplementary material for: 3D Bioprinted Spheroidal Droplets for Engineering the Heterocellular Coupling between Cardiomyocytes and Cardiac Fibroblasts
Source: Cyborg Bionic Syst. 2021 Dec 28;2021:9864212. doi: 10.34133/2021/9864212 (PMC9254634; doi:10.34133/2021/9864212)
Supplement: Supplementary Materials — Table S1: the evolutionary process in engineering a 3D bioprinted spheroid. Figure S1: average aspect ratio (mean ± SD) calculations for the other corresponding generations as enlisted in Table S1. Figure S2: cell viability was assessed by performing the live/dead assay on 2D structures after 4 days of culture. Figure S3: scanning electron microscopy and EDS for cellular encapsulation. Figure S4: shown is an image depicting heterocellular coupling between CM (green) and CF (red) after 21 days of culture (left). All cells (CM+CF) in the presented image are depicted with DAPI (blue) staining (right). Figure S5: shown is a bright field image of CTV dye-stained CM cells. [file 9864212.f1.zip › supplementary information.docx]

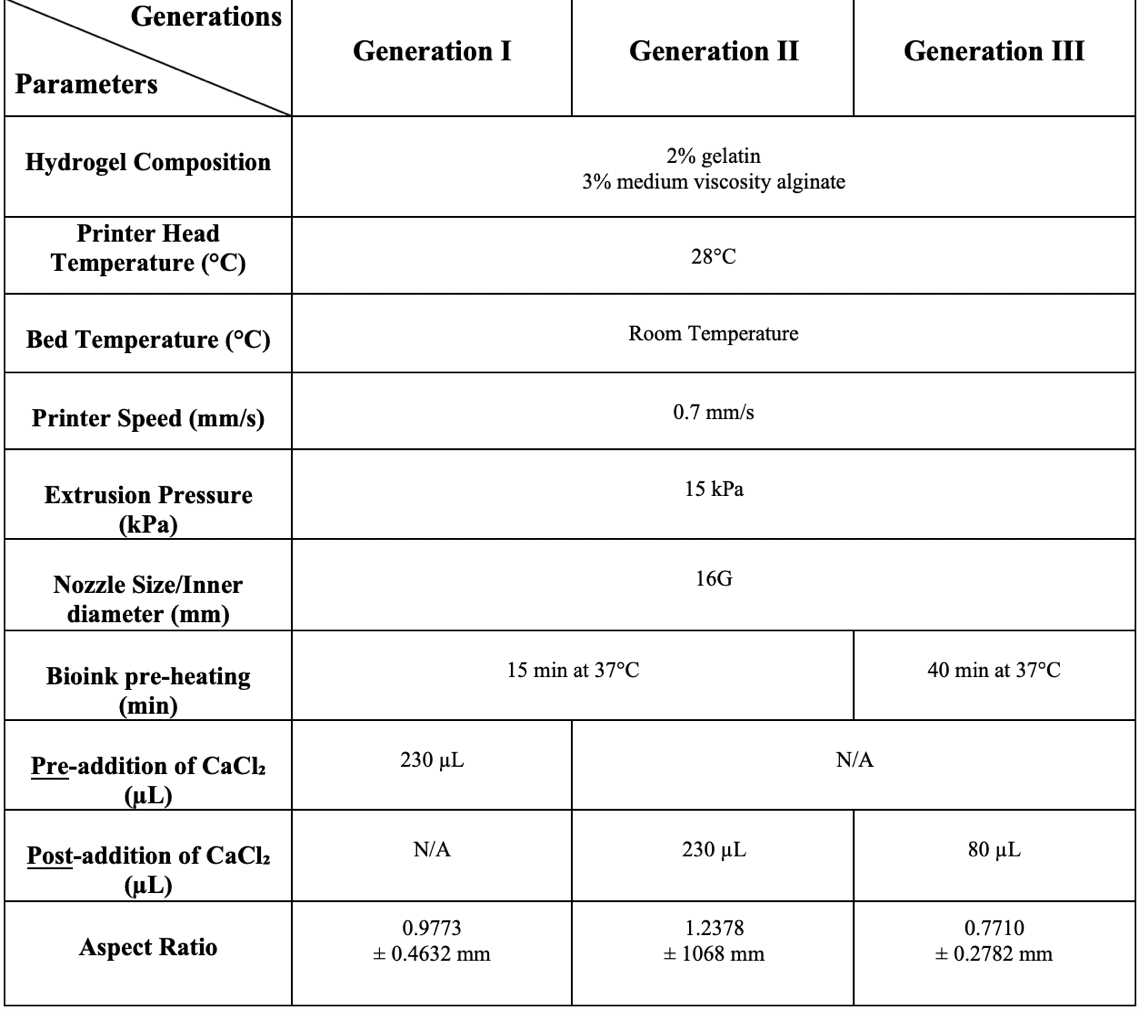
**Table S1:** The evolutionary process in engineering a 3D bioprinted spheroid


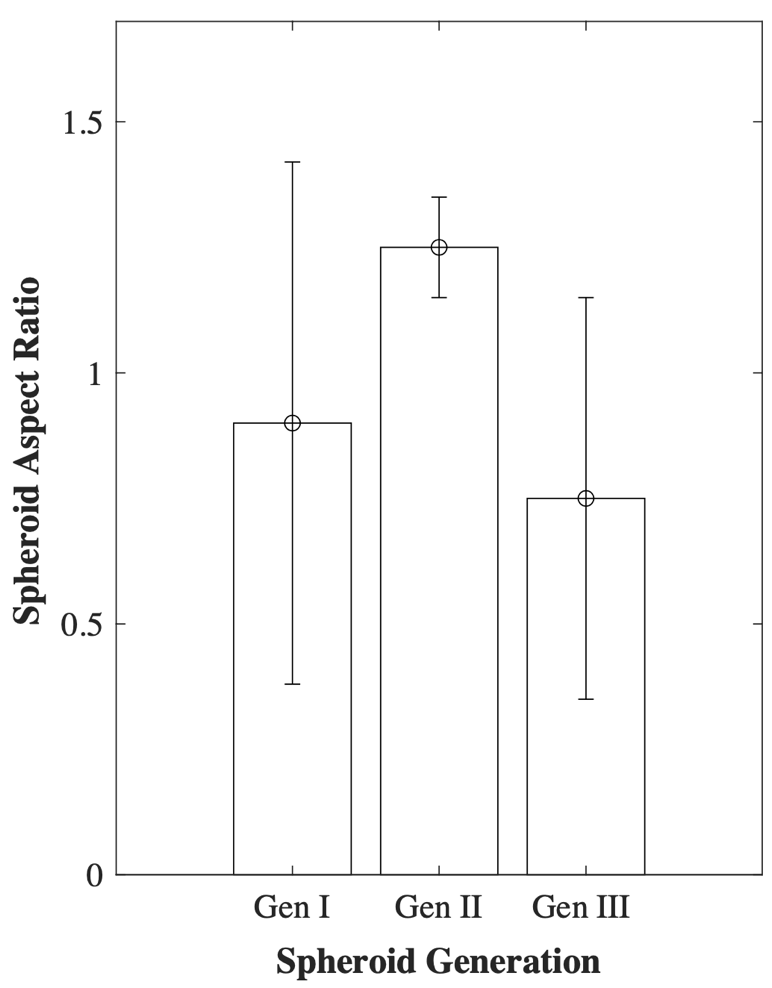


**Figure S1.** Average aspect ratio (mean ± SD) calculations for the other corresponding generations as enlisted in **Table S1**.

**Figure S2.** Cell viability was assessed by performing the live/dead assay on 2D structures after 4 days of culture. (A) Shows live cells cultured atop the spheroids (positive control) and stained in green by calcein AM whereas dead cells are stained in red by ethidium homodimer. (B) Depicts cells cultured in plastic wells (negative control). (C) Represents cells printed in a 3D spheroid stained in red by ethidium homodimer after intended apoptosis with ethanol treatment.

**C**


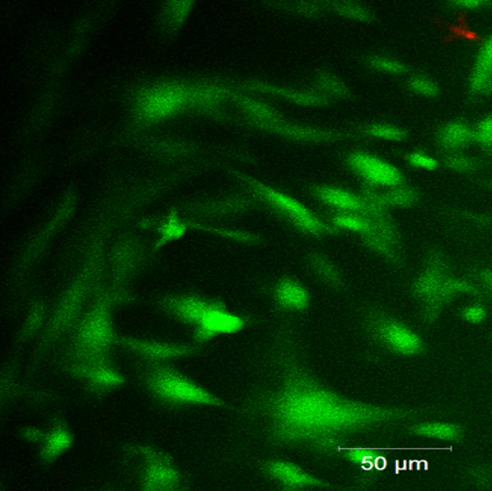

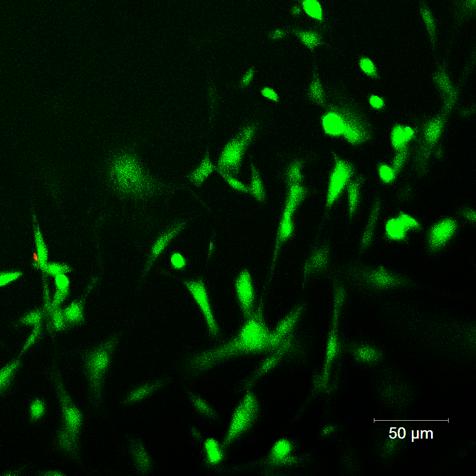


**Positive Control**

**Negative Control**

**A**

**B**

**C**

**Figure S3.** Scanning Electron Microscopy and EDS for cellular encapsulation. Elemental analysis was made using EDS of cell infiltrated surfaces at day 7 identified by SEM (A-B) in comparison with non-cell added scaffolds (image not included). Table below demonstrates a quantitative EDS analysis for the variation of elements detected in acellular vs cellular scaffolds. *p values were found to be all statistically different.

**Figure S4.** Shown is an image depicting heterocellular coupling between CM (green) and CF (red) after 21 days of culture (Left). All cells (CM+ CF) in the presented image are depicted with DAPI (blue) staining (Right).

**Figure S5.** Shown is a bright field image of CTV dye-stained CM cells.
